# An Investigation into the Use of Manufactured Sand as a 100% Replacement for Fine Aggregate in Concrete

**DOI:** 10.3390/ma9060440

**Published:** 2016-06-02

**Authors:** Martins Pilegis, Diane Gardner, Robert Lark

**Affiliations:** Cardiff School of Engineering, Cardiff University, Queens Buildings, Cardiff CF24 3AA, UK; PilegisM@cardiff.ac.uk (M.P.); Lark@cardiff.ac.uk (R.L.)

**Keywords:** manufactured sand, concrete, artificial neural networks

## Abstract

Manufactured sand differs from natural sea and river dredged sand in its physical and mineralogical properties. These can be both beneficial and detrimental to the fresh and hardened properties of concrete. This paper presents the results of a laboratory study in which manufactured sand produced in an industry sized crushing plant was characterised with respect to its physical and mineralogical properties. The influence of these characteristics on concrete workability and strength, when manufactured sand completely replaced natural sand in concrete, was investigated and modelled using artificial neural networks (ANN). The results show that the manufactured sand concrete made in this study generally requires a higher water/cement (w/c) ratio for workability equal to that of natural sand concrete due to the higher angularity of the manufactured sand particles. Water reducing admixtures can be used to compensate for this if the manufactured sand does not contain clay particles. At the same w/c ratio, the compressive and flexural strength of manufactured sand concrete exceeds that of natural sand concrete. ANN proved a valuable and reliable method of predicting concrete strength and workability based on the properties of the fine aggregate (FA) and the concrete mix composition.

## 1. Introduction

In many countries sources of natural sand for use as an aggregate in construction are becoming scarce as sand pits are exhausted and environmental legislation prevents dredging [1,2,3]. This is driving the need to source alternative aggregates, such as those from construction and demolition waste. One possible source of construction aggregate is sand that has been manufactured from the surplus material (crusher dust) that results when coarse aggregate is produced in hard rock quarries. Coarse aggregate production typically yields 25% to 45% crusher dust depending on the parent rock, crushing equipment and crushing conditions [2]. The UK has significant reserves of crusher dust in its quarries, which could undergo further processing to provide the majority of the sand required by the construction industry, using the same sales and delivery channels as it does now for its coarse aggregates. The advantage of this being in the ability to specify aggregates from quarries close to their place of end-use, thereby shortening transport distances and minimising pollution. However, compared to natural sand crusher dusts tend to have inferior shape and texture properties as well as poor grading and unfamiliar mineralogical compositions, all of which affect the properties of fresh and hardened concrete.

The shape and texture of crusher dust depends mainly on (i) the type of crusher [3,4]; (ii) the ratio of the size of material fed into the crusher to the size of the finished product (reduction ratio) and (iii) the parent rock. Impact crushers break rock by “hitting” the material, which causes the rock to break along natural zones of weakness along grain interfaces [5], generally producing particles with good cubical shape. Jaw and large gyratory crushers generally produce particles with poor (non-cubical) shape due to the fact that the crushing chamber is rarely full to permit interparticle crushing [5]. Impact crushers are widely used to crush a range of soft to hard rocks such as basalt, granite, hard limestone. The loading conditions of impact crushers typically lead to a higher probability of fracture of either weak or flaky particles, with fracture occurring by cleavage, with a marked contribution from surface attrition. The result is that more equi-dimensional fine aggregates are produced by this crushing process in comparison to other techniques, such as cone, jaw and roll crushers. It has been shown that the more angular the shape of the fine aggregate, the greater the water demand in concrete and mortars and therefore the use of impact crushers minimises this adverse effect [6]. Nevertheless, researchers have also found that the flexural and compressive strength of concrete benefits from the angularity of crushed fine aggregate due to improved bond and aggregate interlock in comparison to natural sand concretes and mortars at the same w/c ratio [6,7].

The typical particle size distribution of crusher dust rarely conforms to the requirements of national standards. This is mainly due to an excess (>20%) of fine particles passing the 63 µm sieve and a deficiency of particles in the size range 0.3 mm to 1 mm. Crusher dust can produce “harsh” mixes with bleeding problems if it is washed and screened to fall within the prescribed limits. Therefore, to minimise voids and reduce water demand in concrete, crusher dusts are blended with fine natural sand to aid workability and finishability [8]. The particles passing the 63 µm sieve, referred to as fines in this paper, can greatly affect the fresh properties of concrete, as they increase the specific surface area of the fine aggregate, thus requiring an increase in water/admixture dosage for constant workability [7]. If the parent rock is free of clay, then acceptable concretes incorporating 15% to 20% of fines can be produced [1,9]. Conversely, the presence of clay in the parent rock and thus fines can have a detrimental effect on not only the water/admixture demand, but also on the performance of the hardened concrete [10,11]. Thus, it is important to identify an effective and quick method for screening the fines for potentially deleterious particles and establish appropriate limitations for their use in concrete.

Many studies have examined the influence of the partial replacement of fine aggregate in concrete using crusher dusts or small crushed sand samples on concrete properties [8,12,13]. However, little work has been performed on the complete replacement of natural fine aggregate in concrete with crusher dusts. In response to this, this study investigated a range of sands produced by an industry sized KEMCO V7 crushing plant, which reprocesses crusher dusts in a modified impact crusher and classifies the size of the particles with an air screen, as described in more detail by [14]. This process results in a well graded and shaped sand product with a filler component mostly comprising fines. The plant can be viewed as an additional crushing stage in the quarry, which could be used to reprocess surplus crusher dust, therefore increasing the total yield of the quarry.

Since manufactured sands possess different properties to natural sands it would be beneficial to be able to predict the properties of the resultant concrete without extensive laboratory testing. There have been numerous attempts to model the influence of the physical and chemical characteristics of aggregates on the fresh and hardened properties of concrete and provide concrete mix design procedures [15,16]. These, to some extent, take into account a number of the aggregate characteristics: the particle size distribution, maximum aggregate size and aggregate type (natural or crushed). However, as these procedures are based on statistical data from many concrete mixes, the results are generalized and in the case of a specific type of aggregate, like crusher dust or manufactured aggregates, might not yield the expected final concrete properties. Furthermore, the concrete compressive strength estimates are based on the w/c ratio, which for typical aggregates might be correct, but for very angular or very fine aggregates might prove to be an inaccurate representation of the strength. Similar effects might be encountered in consistency measurements.

Several models which evaluate particle packing in aggregate blends have been developed and investigated [17,18,19,20]. It has been concluded that they are useful tools for modelling the aggregate blends with minimum voids contents. However, the most common assumptions in the packing models are that particles are spherical and thus the minimum voids content aggregate and cement combinations do not necessarily lead to the expected concrete mix properties. The Compressible Packing Model [21] has been shown to be relatively accurate with a variety of aggregates, including crushed limestone sands with high filler contents, however, it tends to overestimate the consistency [22] and there is no reference to the effects of clay particles on the fresh and hardened concrete properties.

A number of researchers have turned to ANN models for the prediction of concrete properties using mix composition parameters, for a variety of concrete types [23,24,25,26]. However, these still do not fully take into account the properties of the aggregates. The development of an ANN model which accounts for both the aggregate properties and mix composition could be a useful tool in assessing the expected fresh and hardened performance of concrete made from manufactured aggregates.

The primary purpose of this paper is to present a method by which sands manufactured from crusher dusts may be characterised according to their physical and mineralogical properties and subsequently used as a 100% replacement for natural sand in concrete. The structure of the paper is as follows:

- Section 2 provides experimental details related to the use of a range of manufactured sands of varying fines content as a complete replacement for natural sand in concrete. It also presents the selection and justification of the fine aggregate characterisation tests used in this study.
- Section 3 presents the fresh and hardened concrete test results in combination with the fine aggregate characterization results and uses these to evaluate the properties that make manufactured sand suitable for concrete applications.
- Section 4 describes the development, training and evaluation of an ANN model using the data presented in Section 3 and a further series of validation concrete mixes. The ANN model is used to predict the compressive strength and workability of concrete using the properties of the fine aggregate as one of the main model input variables.

## 2. Materials and Methods

### 2.1. Materials

In this study CEM I 52.5N cement (CEMEX, Rugby, UK) complying with BS EN 197-1:2011 with a chemical composition given in Table 1 was used together with a crushed limestone 4/20 mm coarse aggregate (CA) (CEMEX, Cardiff, UK). The particle size distribution of the latter is reported later in Section 3.1. Where necessary a mid-range water reducing admixture WRDA 90 (Grace Construction Products, Warrington, UK) complying with British Standard BS EN 934-2:2001 was also incorporated.

For this study limestone, granite, basalt and gritstone crusher dusts were processed in the V7 plant. The V7 crushing plant has the ability to produce different sand gradations. At least four manufactured sand gradations from each crusher dust were produced and tested. Also the 0/4 mm fractions of the crusher dusts (unprocessed) were included for comparison. Sea dredged natural sand complying with BS EN 12620:2002 was used as the control fine aggregate. Table 2 shows the notation used in this paper for all the fine aggregates.

### 2.2. Fine Aggregate Tests

As discussed earlier, the grading of the fine aggregate is an important factor that influences the performance of concrete, therefore all fine aggregates were tested for particle size distribution according to BS EN 933-1:1997. The shape and texture of the sand is more difficult to measure directly, hence the predominantly qualitative characterisation tests that are employed in the UK national standards. However, the New Zealand flow cone (NZFC) test (NZS 3111:1986) used in the study offers an indirect measure of shape and texture through measurement of (i) the flow time of fine aggregate through a funnel of known geometry and (ii) the uncompacted voids content of the fine aggregates once it is collected in a receiving chamber. The flow of the material is mostly affected by the shape and surface texture of the particles and the voids content is determined by the grading and shape of the particles [1]. A standard specification envelope described in NZS 3121:1986 for voids content *versus* flow time has been developed to identify the performance of various natural sands in concrete. The envelope is based on the experiences of the New Zealand authorities and is included in this paper for comparison with manufactured sands.

Also as highlighted in the introduction, the presence of deleterious particles like clays may have a detrimental effect on the water demand of the fresh concrete and the performance of the concrete in its hardened state. Therefore, a quick and effective method was required to screen the sands. Two methylene blue (MB) value tests were used; the British Standard test (BS EN 933-9:1999 on the 0/2 mm fraction) involving titration with an MB solution and a test developed by Grace Construction Products (ASTM WK36804) using a pre-calibrated colorimeter allowing a direct estimation of MB solution consumption. These tests will be referred to as the Methylene Blue Value (MBV) and the Grace Methylene Blue Value (GMBV), respectively in this paper. A sand equivalent (SE) test (BS EN 933-8:1999 on the 0/2 mm fraction) was also used, which evaluates the proportion of very fine and clay sized particles in the whole sample. Manufactured and crushed sands will usually have lower SE values than clean natural sands due to the dust of fracture created during the crushing process.

Particle density and water absorption, which are functions of the mineralogical composition of the aggregate, were determined according to BS EN 1097-6:2000. The dry density measurement was used in the NZFC voids calculation, and the absorption capacity was used to adjust the water content of the different concrete mixes.

### 2.3. Concrete Tests, Curing and Specimen Details

The main characterisation parameters of a concrete mix are its workability and strength. The fresh concrete was therefore tested for slump according to BS EN 12350-2:2009. Furthermore, observations were made during mixing, placing and finishing of the concrete specimens as the slump test alone does not fully characterise the workability of the concrete mixes incorporating manufactured sands [8].

Hardened concrete was tested for compressive strength (*f’_c_*) at 1, 7 and 28 days according to BS EN 12390-3:2009 and flexural strength (*f_t_*) at 28 days according to BS EN 12390-5:2009. Standard laboratory moulds were used for the compressive and flexural strength tests. These had dimensions of 100 × 100 × 100 mm^3^ and 500 × 100 × 100 mm^3^ respectively, complying with BS EN 12390-1:2012 mould size requirements with regard to maximum aggregate size. It is acknowledged that using these sizes of specimens for concrete made with 20 mm coarse aggregate may have resulted in a slightly greater variability, and marginally lower strengths, than would have been obtained with larger specimens (e.g., 150 × 150 × 150 mm^3^). This is due to the increased relative heterogeneity and the ‘so-called’ wall effect that occurs when the maximum aggregate size to specimen dimension ratio exceeds a certain limit (of approximately 0.2) [27]. Nevertheless, good consistency was obtained between the multiple samples tested for each mix, which gives confidence in the values obtained as relative strength measures for the different mixes considered. From each concrete mix, nine 100 × 100 × 100 mm^3^ cubes and three 500 × 100 × 100 mm^3^ beams were cast. These were de-moulded after 16 h and placed in a water tank at a temperature of 20 ± 3 °C until the testing age was reached.

### 2.4. Concrete Mix Composition

The study was conducted in two phases. The first phase considered slump-controlled mixes without the addition of water reducing admixtures. A control mix with natural sand was made with a target S2 slump (50–90 mm) as designated by BS EN 206-1:2000. For the manufactured sands, the sands with a -B notation were mixed to achieve the same S2 slump as the control and the required water/cement ratio was recorded. That w/c ratio was then kept constant for the remaining gradings of the same quarry sand, recording the change in slump observed in each concrete mix. The second phase of the study comprised mixes made with a constant w/c ratio (w/c = 0.55) and the incorporation of a sufficient volume of a water reducing admixture to achieve an S2 slump. The water reducing admixture was added in increments of approximately 6 mL and slump tests were repeated until an S2 slump was recorded. The limestone sand mixes achieved an S2 slump at a w/c ratio of 0.55 without admixtures, therefore these were mixed at a lower w/c ratio of 0.50. In order to compare the fresh and hardened concrete properties between mixes, the replacement of the fine aggregate in each mix was performed on a weight by weight basis. This ensured that CA/FA and FA/cement ratios were kept constant. Furthermore, the entrained air content of the mixes was tested according to the pressure gauge method in BS EN 12350-7:2009.

The mix composition is shown in Table 3. In all of the concrete mixes the mass of the water and aggregate was adjusted according to the absorption capacity and water content of the fine and coarse aggregate, in order to maintain a constant w/c ratio by mass for each manufactured sand.

## 3. Results and Discussion

### 3.1. Fine Aggregate Characterisation Results

**Figure 1:**
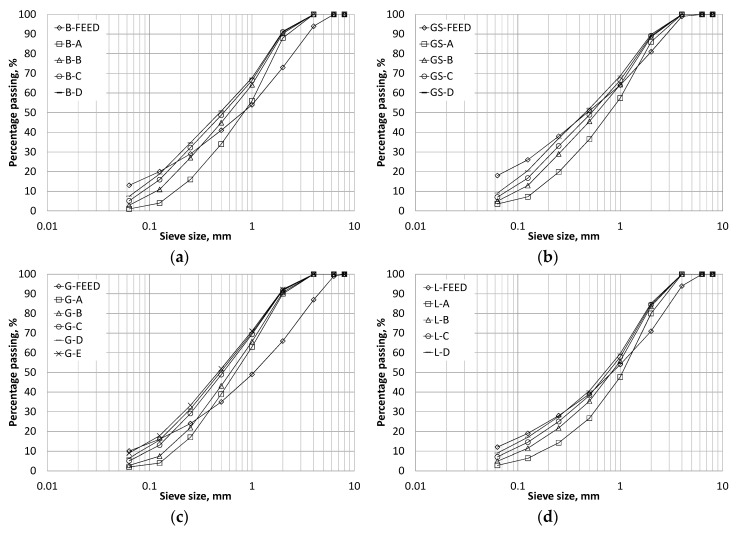
Manufactured sand particle size distributions for (**a**) Basalt sand; (**b**) Gritstone sand; (**c**) Granite sand; (**d**) Limestone sand.

**Figure 2:**
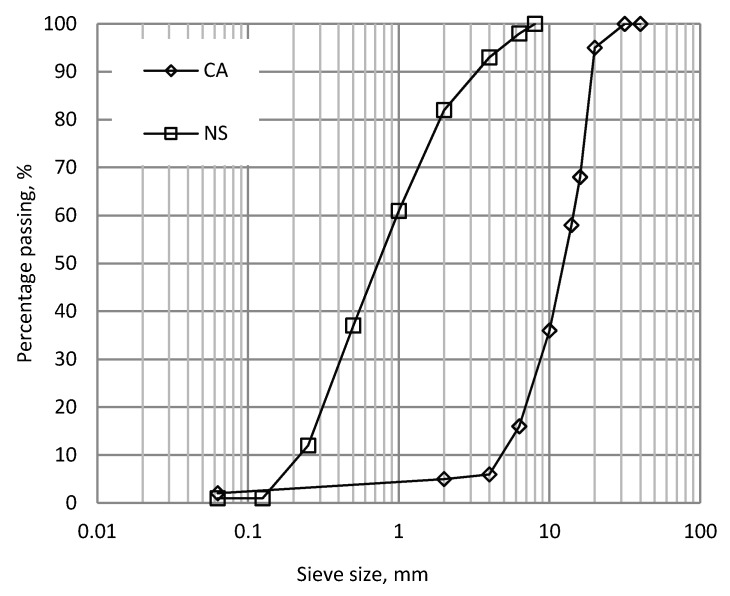
Coarse Aggregate and Natural Sand particle size distribution.

Figure 1a–d shows the particle size distributions of the manufactured sands and corresponding unprocessed crusher dusts, with the coarse and control fine aggregate particle size distributions given in Figure 2. It is evident that the gradings obtained for size fractions above 63 µm for the manufactured sands are all very similar, irrespective of rock mineralogy, and this is a key feature of the KEMCO V7 processing plant. It is assumed that the fines of different mineralogy sands are similar in terms of shape and particle size distribution due to the use of the same production process for all sands.

The fines content of the manufactured sands ranged from 1% to 9% In comparison with the unprocessed crusher dust materials, the majority of the manufactured sands had a greater quantity of the 0.3 mm to 1 mm particles, as indicated by the steeper gradients of the grading curves in this region. The improvement in the particle size distribution in this range was not as pronounced in the limestone sands as in the other manufactured sands. As commented previously this particle size range is often deficient in crushed rock sands [1], therefore necessitating that they are blended with fine natural sands to enable them to be suitable for use in concrete applications. This suggests that, as far as the particle size distribution is concerned, these manufactured sands should prove suitable replacements for fine aggregates in concrete without the requirement for blending with natural sand.

**Figure 3:**
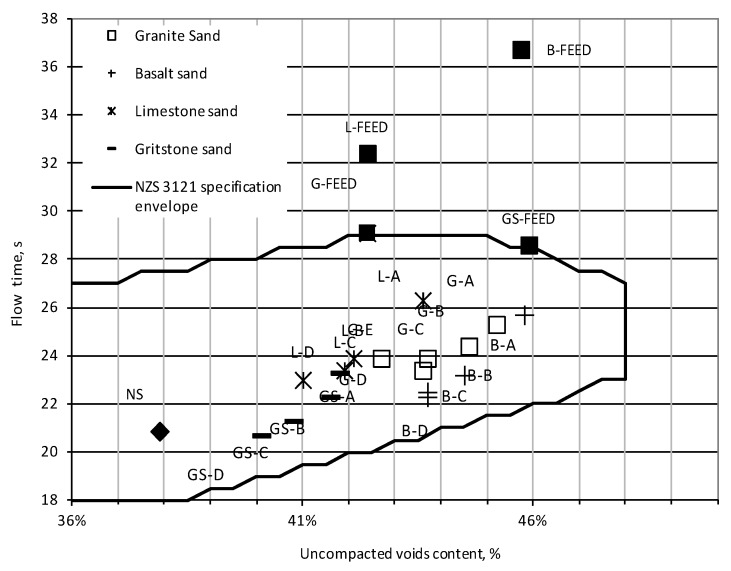
New Zealand flow cone results.

All the manufactured sands used in this study fall within the New Zealand standard envelope for natural sands unlike their crusher dust counterparts, as observed in Figure 3. This suggests that the grading and shape of the manufactured sands should be suitable for use in concrete applications.

**Figure 4:**
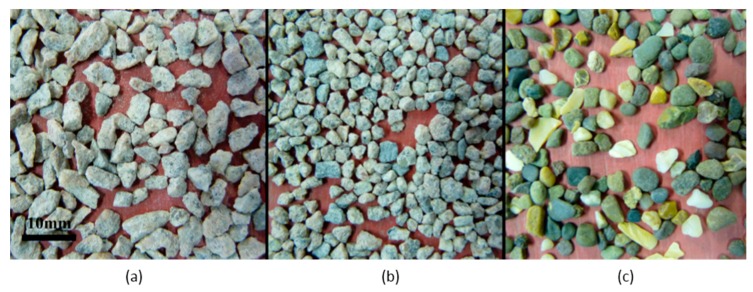
Images of 4–2 mm sand fractions (**a**) G-FEED (**b**) G-A (**c**) NS.

As shown in Figure 4 the natural sand particles were smooth and rounded, whereas the unprocessed crusher dusts comprised flat and elongated particles, which were angular with sharp edges. The manufactured sands were again angular but were more equi-dimensional and rounded than the unprocessed crusher dusts. The images in Figure 4 are typical for all fractions and types of crusher dusts and manufactured sands used in the study. The NZFC results confirm that the smoother and rounder the sands the lower the flow time.

Assuming that the governing factor for the flow time measurement is the shape and surface texture of the fine aggregate then there is evidence of the KEMCO V7 process improving these characteristics as the flow time of the unprocessed crusher dust material was between 28 s and 37 s, whereas for all the manufactured sands it was in range of 21 s to 27 s. If processed sands with a particular mineralogy are considered, it can be seen that flow times were slightly reduced with an increase in fines content. This reduction was typically 1 s to 3 s for the -D gradings. It can therefore be inferred that a major part of the flow time reduction can be attributed to particle shape improvements due to processing.

The un-compacted voids content of all the basalt and gritstone sands was below that of their feed material. However, the voids content of all the granite sands and the coarsest limestone sand (L-A) was greater than that of their corresponding crusher dusts. This can be attributed to the combined effects of changes in grading as well as the shape of the particular sands.

**Figure 5:**
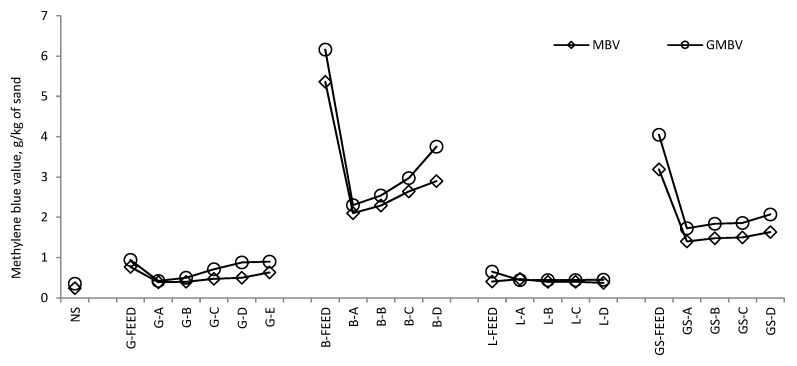
Methylene blue value and Grace rapid clay test results.

MBV and SE tests were used to identify the presence of potentially deleterious particles, and in particular clays, in the fine aggregates under investigation. Figure 5 shows that the natural, granite and limestone sands had low MBV, less than 0.63 g/kg, whereas the basalt and gritstone sands had MBV above 1.73 g/kg. This suggests the presence of clays in the basalt and gritstone sands which may result in an increased water and admixture demand when used in a concrete mix [11]. However, it is possible via the air classification stage in the KEMCO V7 manufacturing process to remove a portion of the deleterious fines, as demonstrated by the reduction in the MBV for all sands compared to their unprocessed crusher dust counterparts. For the basalt and gritstone sands the MBV increases with an increase in fines content as a result of a greater quantity of clay particles in the fines fraction, whereas the marginal increase observed in the MBV for the granite and limestone sands is due to the slight increase in the specific surface area of the fines fraction. Standard MBV and GMBV tests show a direct correlation and because the latter is quicker than the standard MBV test it would seem to be a valuable and reliable alternative.

**Figure 6:**
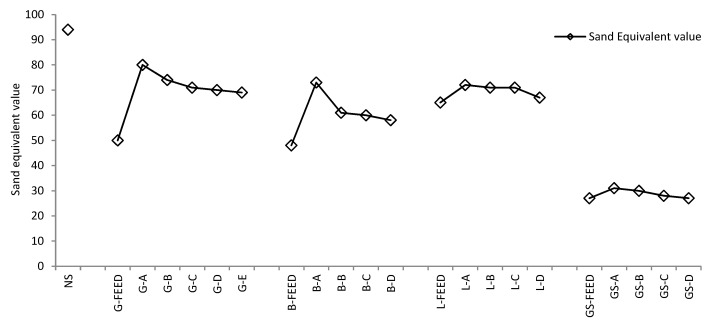
Sand equivalent value for fine aggregates.

Figure 6 shows that the natural sand had the highest SE value of 92 followed by the granite and limestone sands, which had SE values in the range of 67 to 80, basalt sands (58 to 73) and gritstone sands (from 27 to 31). As observed in the MBV test results, for a particular mineralogy of sand, the SE values decreased as the amount of fines increased. However, the MBV and SE test results did not show a direct correlation, as is also reported by Nikolaides *et al.* [28]. This could be due to the SE test being more sensitive to the proportion of dust of fracture than the MBV test.

It can be concluded that for parent rocks containing clay the manufactured sand will also include a proportion of clay in the fines, whereas if the parent rock is clean then the fines are the dust of fracture created during the processing.

The water absorption (WA24) of the unprocessed crusher dusts was either higher or the same as that of the corresponding manufactured sands as shown in Table 4. Processing of the crusher dust might have induced fracture of particles through water accessible voids. This in turn could have reduced the number and volume of these voids which are measured by the water absorption test [29]. Furthermore, BS EN 933-1:1997 states that the tested sands must be washed over a 63 μm sieve, but coatings like clays may not be readily removed by washing, resulting in higher absorption values for aggregates with higher initial fines contents. This assumption is supported by the highest water absorption values being found for sands with high MBV. The dry density was found to be relatively constant for the unprocessed crusher dust and their corresponding manufactured sands.

### 3.2. Concrete Results

**Figure 7:**
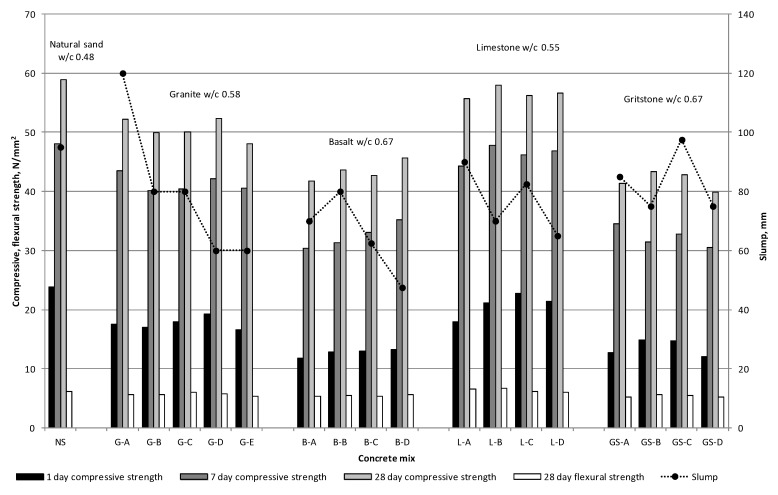
Slump controlled mix results.

**Figure 8:**
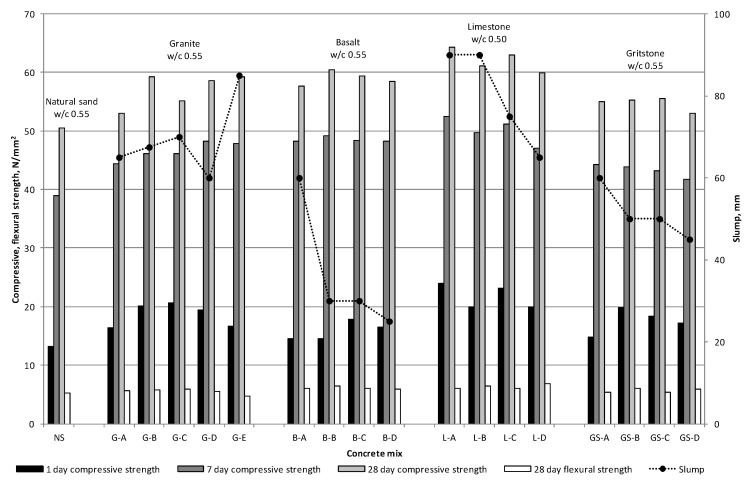
Water/Cement ratio controlled mix results and admixture dosages.

The compressive strength of the slump controlled mixes was predominantly governed by the w/c ratio, as observed in Figure 7. The lowest w/c ratio of 0.48 for natural sand yielded the highest 28 days compressive strength, closely followed by the limestone, granite, basalt and gritstone sands. At the same w/c ratio of 0.55 the compressive strength of all manufactured sand mixes exceeded that of the natural sand control (Figure 8), whilst being comparable to that of the slump controlled natural sand mix. Similarly, the flexural strength of all the mixes except the G-E mix exceeded that of the natural sand control when mixed at constant w/c ratio. The compressive and flexural strengths of the limestone mixes at a w/c ratio of 0.50 exceeded or were equal to that of the control natural sand mix.

The granite, limestone and natural sands with low MB values had much lower water and water admixture demands to achieve the S2 slump than the gritstone and basalt sands with MBV above 1.73, as seen in Figure 7 and Figure 8. This confirms the usefulness of MB tests in identifying fine aggregates which could detrimentally affect the fresh properties of concrete due to presence of clay particles.

These results suggest that the angular shape and rough surface texture of the manufactured sand contributed to the compressive and flexural strength of the mix by virtue of aggregate interlock and improved bond between the cement matrix and aggregate particles. Similar results have been obtained by other researchers [5,8]. At w/c ratio of 0.55 all manufactured sand mixes at 28 days attained compressive strength in the range of 53 to 60.5 N/mm^2^. This suggests that the particular clays present in the gritstone and basalt sands do not negatively affect the 28 days strength at the same w/c ratio. However, the long term effects of MB value and clays on strength were not investigated in this study.

Some of the basalt and gritstone sands mixed at a w/c ratio of 0.55 did not achieve the S2 slump even with admixture dosages exceeding those recommended by the manufacturer. This suggests that if a workable concrete mix with reasonable w/c ratio is required then the presence of clay in the manufactured sand is the first and foremost limiting factor. However, if high w/c ratios are permissible for the given concrete application, then an increase in the w/c ratio can offset the negative effects of the presence of clay on the workability of the concrete.

Table 5 reports the entrapped air and admixture dosage for both phases. The entrapped air measurement from phase 1 ranges from 0.45% to 1.60%. Within each concrete mix it is relatively constant with the exceptions of the G-B and GS-B mixes. This may be attributed to either excessive or insufficient vibration of these concrete samples respectively. In phase 2 the entrapped air measurement ranges from 0.80% to 1.80%. Again, within each concrete mix the measurement is relatively constant and slightly elevated in comparison to phase 1 results. This could be due to both the entrainment of air as a result of the incorporation of a plasticiser as well as due to reduced consistency, which may result in a higher number of air voids in the hardened concrete.

A general trend of decreasing slump with increasing fines content was observed in the majority of mixes made without the use of an admixture, with the exception of the B-B, L-C and GS-C mixes, which exhibited higher slump values. This may be explained by the lubricating effect of the increased fines content [30]. However, a further increase in the fines content offsets this benefit due to an increase in the specific surface area of the aggregate that has to be covered by paste to provide the same workability. However, for the granite and limestone sands the decrease in slump with increasing fines content at constant w/c ratio still resulted in a slump within the S2 slump range. This suggests that clay-free fines contents ranging from 1% to 9% for a particular mix design do not have a significant effect on the slump of the concrete. For a constant slump value an increase in fine aggregate fines content would generally demand higher admixture volumes. However, since an S2 slump range was targeted in phase 2, some variation in the admixture dosage is to be expected, as observed for the Gritstone and Limestone sands in Table 5. The lubricating effect of the fines as discussed for the phase 1 mixes is also applicable to the phase 2 mixes.

During the mixing and casting stages it was observed that the natural sand mix was the easiest to handle and finish as expected due to the rounded and smooth fine aggregate. Mixes with -A notation were sometimes “harsh” and could only be finished with some effort due to the low amount of fines and lack of particles with diameters less than 1 mm. Mixes G-B, G-C, G-D, L-B and L-C with low MBV were found to be easy to handle and finish even though they had lower slump values than the -A mixes. Gritstone mixes at a w/c ratio of 0.67 were found to be easy to work and finish. The basalt mixes were quite cohesive but finished well. The B-D mix was found to be very cohesive and rapidly lost workability, which could be attributed to the absorption of water from the mix by clay present in the high fines content. If the gradings in Figure 1a–d are considered, it can be concluded that for manufactured sand the clay-free fines content together with the presence of particles smaller than 1 mm are important for providing adequate handling and finishing properties of concrete.

No noteworthy correlation between compressive strength, flexural strength and the fines content or the grading of the fine aggregates was observed, suggesting that in the range 1%–9%, the fines do not significantly affect the compressive and flexural strength for a given mix composition, when admixtures are employed to enhance workability. The importance of considering other methods to address the lack of fines such as the use of filers and supplementary cementitious materials, as well as the influence of cement content on the performance of mixes containing manufactured sands, is fully acknowledged and is the subject of ongoing work. The results imply that higher levels of fines may be employed in concrete applications with associated reductions in the storage or disposal of the fines material. Additional benefits of enhanced durability due to the pore blocking nature of the fines material (as reported by [12,31]) may also be realised through the use of higher fines contents, although this is outside the scope of this paper.

## 4. Artificial Neural Network Modelling

ANN modelling was selected for this study due to its ability to generalize multiple variable, non-linear, complex relationships and thereby predict results from a range of inputs with which they have been trained [32]. In this paper Multiple Back-Propagation (MBP) version 2.2.4 [33], a free software package was used to build and train the ANN models. The obtained connection weights of trained models were then transferred into MS Excel spreadsheets. These were used for prediction analysis, evaluation and comparison of the models.

### 4.1. Choice of Input Parameters

As discussed, the properties of concrete depend on the properties of the aggregates and the mix composition. Since all of the mixes used in this study had the same amount of cement, FA and CA, the only variables were the w/c ratio and water reducing admixture dosage. These were therefore selected as the two input parameters that described the changes in the mix composition. There are three major fine aggregate properties that affect the workability and strength of the concrete, giving a further 8 input parameters: grading (% fines, NZFC flow time and voids content), shape and texture (as inferred by NZFC flow time and voids content), quality of fines (water absorption, GMBV and SE).

### 4.2. Dataset

The model was developed using the data presented in this paper together with that from other similar mixes created in the laboratory during the project giving a total of 44 data entries. These were randomly divided into 35 training and 9 testing entries. Table 6 shows the range of the input and output values used in the training dataset. The average values represent the properties of a fine aggregate which could be encountered and could be a 50:50 blend of dredged natural sand and clay contaminated quarry dust mixed in concrete with medium dosage of plasticiser and w/c ratio of 0.62.

### 4.3. Model Setup

**Figure 9:**
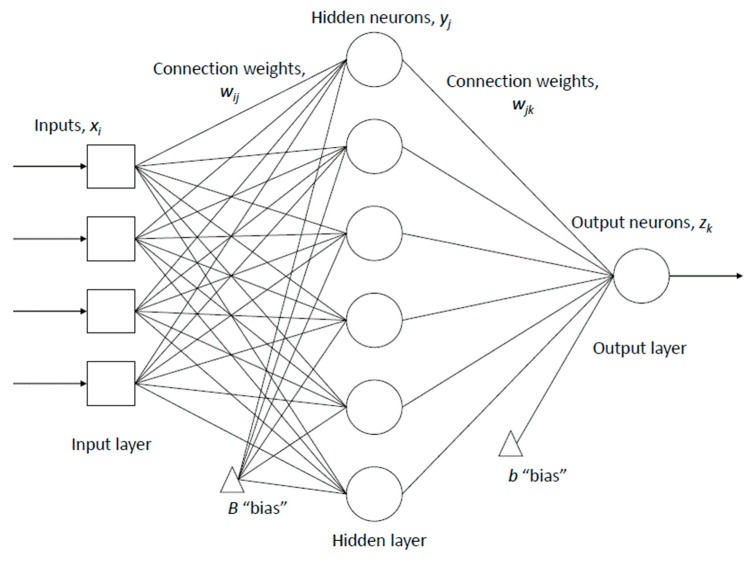
Artificial neural network structure diagram.

A back propagation algorithm was used to train the neural networks. A full description of the algorithm and ANN in general is provided by Fausett [34]. Figure 9 shows the typical structure adopted for these models. After the training of the ANN the resultant connection weights and “biases” were transferred into spreadsheets. In these, Equation (1) was used to calculate the numerical values of the neurons in the hidden layer:

(1) $$y_{j}=F\left(\overset{n}{\underset{i=0}{\sum }}\left(x_{i}\cdot w_{ij}\right)+b\right)$$

where *y_j_* is a neuron in the hidden layer, *x_i_* is the scaled input value, *w_ij_* is the connection weight, *n* is the number of inputs and *b* is a constant referred to as “bias” or “threshold” that is calculated during the network training similarly to connection weights. *F* is the sigmoid activation function obtained from Equation (2) which represents the non-linear behaviour of concrete. The output values *z_k_* are calculated using Equation (1) but by exchanging *x_i_* with *y_j_* and *w_ij_* with *w_jk_*.

(2) $$F\left(x\right)=\frac{1}{1+e^{-x}}$$

The neural networks were created with a single hidden layer as it has been previously demonstrated to successfully model concrete strength and workability [32,35,36]. The choice of the number of neurons in the hidden layer is a function of problem complexity and is usually determined empirically. Networks with a range of hidden neuron numbers were created and trained using the training data. The prediction errors, in this case the root mean square error (RMS), of the testing data were evaluated and the model with the least error was adopted. Four neural networks with 2, 4, 6 and 8 hidden neurons were created and trained for each output. In this paper neural network models are designated according to the number of neurons in each layer “input layer—hidden layer—output layer” and the output parameter considered (either strength or slump).

The weights for each neuron were randomized before the network was trained. The initial learning rate was 0.7, which was decreased by 1% after 7 learning cycles. In addition, the initial momentum factor was 0.7, which was decreased by 1% after each 500 cycles. The online training mode was used where the weights were updated after each entry and the data was presented in a random order. The training of each network was halted after 5000 cycles. It was observed that the RMS had stabilized for all networks after approximately 2000–3000 learning cycles.

### 4.4. Model Evaluation

Table 7 shows the RMS prediction errors of the models for the testing dataset. It can be seen that the least error for slump prediction is the 8-2-1 slump model, whereas for strength prediction, the least error is obtained with the 8-6-1 strength model, and therefore these were adopted as the most accurate models for the given dataset.

In order to check the prediction capabilities of the models four concrete mixes were made with the same FA, CA and cement contents as detailed in Section 2 but with varying w/c and admixture dosage. These mixes included a crusher dust which was not used in the training or testing of the models, natural sand, granite sand without clays and gritstone sand with clay particles. The model input values taken from the validation mixes are given in Table 8.

**Figure 10:**
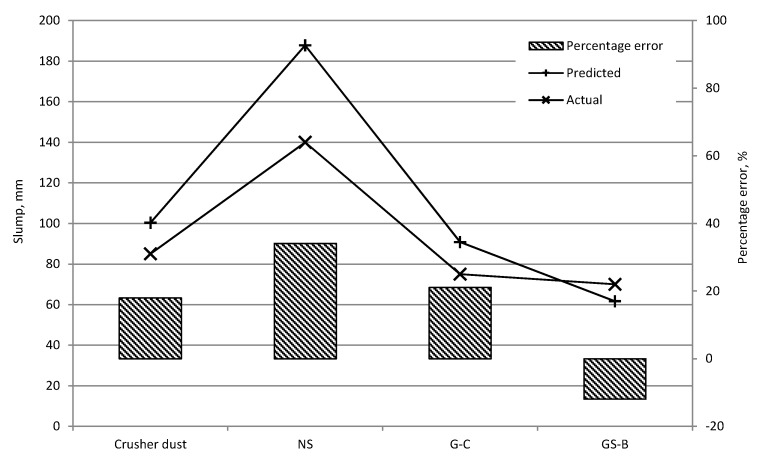
Predicted and actual slump values for validation mixtures.

**Figure 11:**
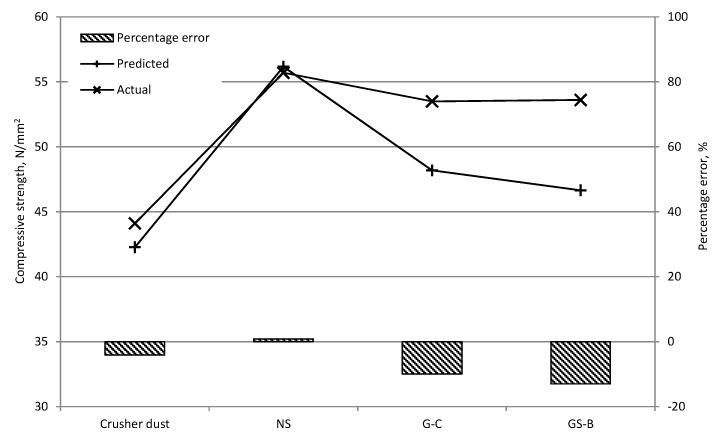
Predicted and actual compressive strength values for validation mixtures.

The predicted and actual slump and compressive strength values for the validation mixtures are shown in Figure 10 and Figure 11 respectively. The 8-2-1 slump model had a RMS of 26.61 mm and highest percentage error of 34% for the NS mix. It has to be noted that in the 50–100 mm slump range, where most of the training data was located, the percentage error does not exceed 21%. Furthermore, taking into account the artificial 300 mm values adopted for the slump collapse in the training data it might be expected that there would be an overestimation of slump in the higher workability mixtures. Nevertheless, an overestimation in slump value is preferred to an underestimation since other techniques may be employed to achieve the desired mix workability. It can be seen that the compressive strength predictions are relatively accurate with a highest percentage error of 13% and RMS of 4.47 N/mm^2^ for the 8-6-1 strength model. Again, an underestimation (if any) in the predicted compressive strength is preferable to an overestimation, particularly in structural design applications.

**Figure 12:**
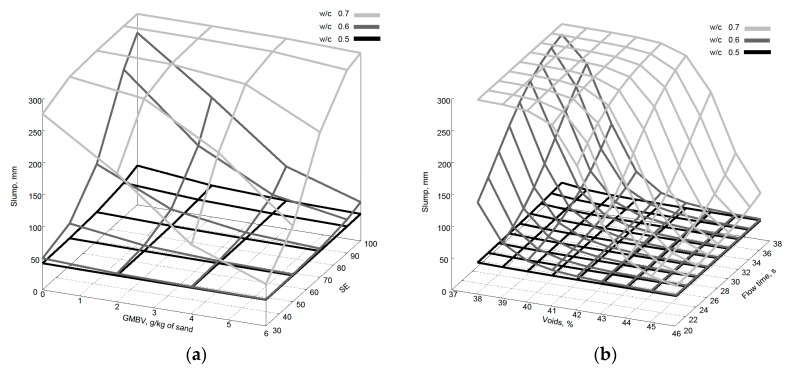
Variation of slump predictions with (**a**) change of SE and GMBV and (**b**) change in voids content and flow time.

Numerical evaluation of the model can help to confirm that the ANN has actually learnt the underlying theoretical relationship. Fine aggregate parameters should be considered concurrently since it is difficult to identify the influence of any one input parameter on the workability and compressive strength of concrete due to the multi-variable, non-linear nature of the relationship between the variables. Figure 12a,b show the variation of slump predictions with SE and GMBV, and flow time and voids values, when all other properties are kept at average values from the training dataset (Table 6). It can be seen that for fluid mixes with w/c ratios of 0.6 and 0.7 there is a prominent decrease in slump as the clay content increases. Whereas in stiff mixes (0.5 w/c ratio) there is little effect on the slump predictions. Similarly, for stiff mixes there is no effect of shape and grading on the slump whereas for fluid mixes the more angular the aggregate as indicated by increase in voids content the lower the slump, similarly the finer the grading, as indicated by decrease in the flow time, the lower the predicted slump.

**Figure 13:**
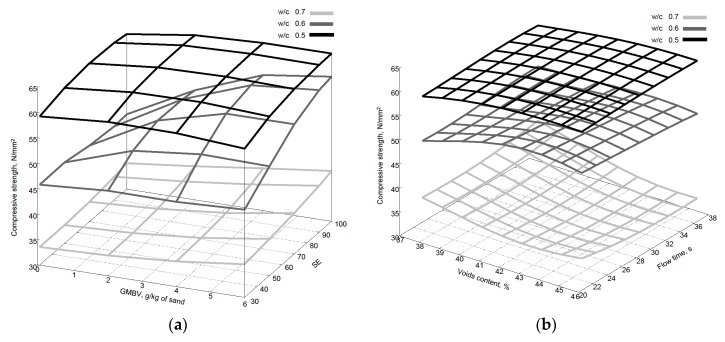
Variation of compressive strength predictions with (**a**) change of SE and GMBV and (**b**) change in voids content and flow time.

Figure 13a,b shows the compressive strength variation due to both SE and GMBV values and voids content and flow time. It can be seen that for SE and GMBV when the w/c ratio is 0.7 the compressive strength predictions are relatively constant whereas for w/c ratio of 0.6 and 0.5 there is an optimum range of values for SE and GMBV that result in the highest compressive strength. Remembering that the w/c ratio is the dominant factor regarding concrete strength, it can be seen that for high w/c ratio (0.7) the shape, texture or grading of the aggregate has little effect on compressive strength in contrast to high strength (low w/c ratio) concretes, as observed by Donza *et al.* [37]. The flow time is mainly defined by the grading and surface texture of the fine aggregate. It has been shown by Li *et al.* [30] that if the grading is the same, then increased flow time indicates a rougher fine aggregate particle surface which increases the compressive strength.

It can be concluded that ANN models can be used to estimate workability and compressive strength of concrete when the fine aggregate properties are used alongside the mix composition as ANN model input parameters. However, there are limitations of such models, the main being that they only perform well in the range of input and output variables with which they have been trained. It can also be concluded that the ANN models developed in this study are valid and the predictions generally correspond to the theoretical relationships between the mix composition, fine aggregate parameters and concrete properties. Thus, this type of model could be used to reduce the effort needed to develop working concrete mixes or to compare the performance of different fine aggregates through simple aggregate classification tests.

## 5. Conclusions

The primary aim of this study was to present a method by which sands manufactured from crusher dusts may be characterised according to their physical and mineralogical properties and investigate their use in concrete as a 100% replacement for natural sand. The results from the experimental study presented in this paper demonstrated that the V7 crushing plant is capable of producing manufactured sands with similar particle size distributions irrespective of the parent rock mineralogy. A series of tests were proposed, which allowed indirect measurement of shape, surface texture, grading and the presence of deleterious fines, which were used to characterise the physical properties of natural sands, unprocessed crusher dusts and manufactured sands. From these tests it was apparent that manufactured sand particles of improved shape and grading compared to natural sand and unprocessed crusher dust were produced by the V7 crushing plant, along with a reduction in the quantity of clay particles in the fines portion compared to the feed material.

Workable concretes were produced using manufactured sand as the sole fine aggregate at different w/c ratios. The presence of clays in manufactured sands can be a limiting factor in their use in concrete applications where high consistency and relatively low w/c ratios are specified. Nevertheless adequate concrete incorporating manufactured sand as sole fine aggregate can be made. Indeed, at the same w/c ratio, the compressive and flexural strengths of manufactured sand concretes were higher than those of their natural sand counterparts. This is believed to be due to the angular shape of the material, which has a positive effect on aggregate interlock and hence leads to improved bond between the cement and aggregate particles. The presence of clay did not affect the 28 days strength of concrete made at the same w/c ratio with differing manufactured sand mineralogies. Therefore, there is the potential for clay containing aggregates to be used in concrete that were previously discouraged. An optimum fines content of 7% was observed to aid the handling, placing and finishing of manufactured sand concrete. However, no significant trend between fines content and concrete compressive strength was observed in the 1% to 9% fines content range that was investigated. Thus, in order to maximise the efficient use of materials, higher fines content manufactured sand gradings could be employed when compressive strength is the control property.

It has been shown that ANN models can be used to estimate the compressive strength and workability of concrete based on the fine aggregate properties and mix composition with reasonable accuracy. Such models, together with the characterisation tests described previously, could be employed by the construction industry when new fine aggregates sources enter the market. The use of the models would eliminate the need for extensive laboratory trials to select an appropriate mix design and establish the fresh and hardened properties of the resulting concrete.

## Figures and Tables

**Table 1:** Cement properties.

Oxide	Oxide Composition (wt %)
SiO_2_	19.7
Al_2_O_3_	4.8
Fe_2_O_3_	3.1
CaO	63.6
MgO	1.2
SO_3_	3.6
Cl^−^	0.1
Free CaO	2.3
Na_2_Oeq ^1^	0.7
LOI ^2^	2.7

^1^ Na_2_O equivalent; ^2^ Loss on ignition.

**Table 2:** Fine aggregate notation.

Description	Fines Content ^1^	Type	Notation
Sea dredged natural sand	1.0	Natural	NS
Basalt crusher dust	10.0	Crushed	B-FEED
Basalt sand	1.0	Manufactured	B-A
Basalt sand	2.9	Manufactured	B-B
Basalt sand	5.1	Manufactured	B-C
Basalt sand	7.4	Manufactured	B-D
Granite crusher dust	13.0	Crushed	G-FEED
Granite sand	2.0	Manufactured	G-A
Granite sand	2.9	Manufactured	G-B
Granite sand	5.1	Manufactured	G-C
Granite sand	6.5	Manufactured	G-D
Granite sand	9.0	Manufactured	G-E
Limestone crusher dust	12.0	Crushed	L-FEED
Limestone sand	2.8	Manufactured	L-A
Limestone sand	4.9	Manufactured	L-B
Limestone sand	7.1	Manufactured	L-C
Limestone sand	9.0	Manufactured	L-D
Gritstone crusher dust	18.0	Crushed	GS-FEED
Gritstone sand	3.5	Manufactured	GS-A
Gritstone sand	5.0	Manufactured	GS-B
Gritstone sand	7.0	Manufactured	GS-C
Gritstone sand	9.0	Manufactured	GS-D

^1^ percent of fine aggregate.

**Table 3:** Concrete mixture composition.

Cement (kg/m^3^)	FA (kg/m^3^)	CA (kg/m^3^)	w/c Ratio	Admixture (L/m^3^)
350	753	1040	Varies ^1^ 0.55 ^2^	0 ^1^ Varies ^3^ (refer to Section 3.2)

^1^ Phase 1—slump controlled mixes; ^2^ Phase 2 limestone w/c = 0.5; ^3^ Phase 2 w/c controlled mixes.

**Table 4:** Water absorption and dry density results.

Aggregate Property	NS	G-FEED	G-A G-B G-C G-D	B-FEED	B-A B-B B-C B-D	L-FEED	L-A L-B L-C L-D	GS-FEED	GS-A GS-B GS-C GS-D
WA24, %	1.04	0.58	0.58	1.92	1.67	0.62	0.45	1.53	0.98
ρ_rd_, Mg/m^3^	2.63	2.62	2.61	2.83	2.87	2.85	2.85	2.64	2.57

**Table 5:** Entrapped air and admixture dosage for Phase 1 and Phase 2 mixes.

Mix Notation	Phase 1	Phase 2	
	Entrapped Air (%)	Entrapped Air (%)	Admixture Dosage (L/m^3^)
NS	0.50	0.90	0.00
G-A	0.45	1.50	0.00
G-B	1.60	1.40	0.00
G-C	0.90	1.30	1.25
G-D	0.65	1.40	0.62
G-E	0.78	1.40	1.00
B-A	0.50	1.41	2.75
B-B	0.50	1.60	2.75
B-C	0.45	1.30	3.30
B-D	0.65	1.80	3.30
L-A	1.40	1.30	1.63
L-B	1.50	1.30	1.10
L-C	1.48	1.10	1.35
L-D	1.38	0.80	1.10
GS-A	1.40	1.28	2.45
GS-B	0.78	1.30	2.75
GS-C	1.00	1.35	2.75
GS-D	1.20	1.56	2.75

**Table 6:** Range of input and output variables and the parameter that they influence.

Variable	Minimum	Maximum	Average	Influence
w/c ratio	0.48	0.75	0.62	Mix composition
Admixture (L/m^3^)	0	3.3	1.65	Mix composition
GMBV (g/kg of sand)	0.35	6.16	3.26	Quality of fines
SE	27	94	60.5	Quality of fines
Water absorption (%)	0.45	1.92	1.19	Quality of fines
Voids (%)	37.9	45.9	41.9	Grading, shape & texture
Flow time (s)	20.7	36.7	28.7	Grading, shape & texture
Fines (% of FA)	1	18	9.5	Grading
28 days *f’_c_* (N/mm^2^)	31.3	64.3	–	Result
Slump (mm)	25	300 ^1^	–	Result

^1^ assumed value for collapse/shear as no value is recorded according to BS EN 12350-2:2009.

**Table 7:** ANN model RMS errors for testing dataset.

Model	RMS (mm)	Model	RMS (N/mm^2^)
8-8-1 slump	13.36	8-8-1 strength	2.70
8-6-1 slump	13.58	8-6-1 strength	2.61
8-4-1 slump	11.50	8-4-1 strength	2.87
8-2-1 slump	7.97	8-2-1 strength	4.09

**Table 8:** Validation mixture input values.

Validation Mix	w/c Ratio	Admixture (L/m^3^)	GMBV (g/Kg of Sand)	SE	Voids (%)	Flow Time (s)	Water Absorption (%)	Fines Content (%)
Crusher dust	0.65	0	1.55	44	42.2	36.6	0.77	9.3
NS	0.51	0	0.35	94	37.9	20.9	1.04	1.0
G-C	0.60	0	0.71	71	43.7	23.9	0.58	5.1
GS-B	0.60	3	1.84	30	41.6	22.3	0.98	5.0
